# Scientific research ability of specialist nurses in Guangxi Zhuang Autonomous Region, China: A cross‐sectional study

**DOI:** 10.1002/nop2.1868

**Published:** 2023-06-19

**Authors:** Ziwei Huang, Yuanfang Liu, Yi Lei, Yiping Wei, Xiaomei Chen, Yuansong Lan

**Affiliations:** ^1^ Nursing department Guangxi Medical University Cancer Hospital Nanning China; ^2^ Department of Basic Medical Research Guangxi Medical University School of Nursing Nanning China; ^3^ Department of Nursing Renji Hospital, School of Medicine, Shanghai Jiaotong University Shanghai China; ^4^ School of Nursing Guangxi Health Science College Nanning China

**Keywords:** cross‐sectional survey, evidence‐based practice, influencing factors, nursing research capacity, specialist nurses

## Abstract

**Aim:**

This study investigated the scientific research ability of Chinese specialist nurses (SNs) in the Guangxi Zhuang Autonomous Region and its influencing factors.

**Design:**

A cross‐sectional design.

**Methods:**

A total of 652 SNs in the Guangxi Zhuang Autonomous Region were investigated from March to October 2021. The nursing scientific research ability level was measured using the Nursing Research Competence of Nurses Self‐evaluation Scale. Descriptive statistics, univariate analysis and ordinal logistic regression analysis were used to evaluate factors affecting the scientific research ability of SNs.

**Results:**

The median score of scientific research ability of SNs was 31 (interquartile range: 19–41). Approximately 74.8% of clinical speciality nurses had low scientific research ability. Educational background, working hospital level, being the first author of a published paper and successful application for scientific research projects were identified as factors influencing scientific research ability score.

## 1 INTRODUCTION

Clinical nurse specialist (CNS) refers to the Registered Nurse (RN) who has obtained the corresponding specialist nurse (SN) qualification certificate after systematic training and examination; has profound nursing theoretical knowledge, expert nursing skills and rich clinical experience in a specific nursing field; and can skilfully use specialist nursing knowledge and skills to provide professional nursing services for patients (Chen & Li, 2015). Nursing scientific research ability refers to the ability of nursing staff to continuously discover general laws and explore truth in the field of nursing. It is an important means to explore nursing theory, nursing method, and nursing technology innovation and improve the quality and efficiency of nursing work (Gao, 2013). The scientific research level and ability of SNs determine whether they can find, analyse and solve clinical nursing problems from a specialist perspective in clinical nursing work, and play the role of nursing experts in practice (Mo et al., 2020a, 2020b).

## 2 BACKGROUND

Nursing research refers to the use of scientific methods to explore clinical nursing practice, evaluation, management and education (Polit & Beck, 2017). It is an important part of professional nursing. Research results can be used to develop and change nursing practice. Evidence‐based medicine has been advocated in the clinical environment for many years and has been widely accepted in nursing and related medical fields (Mackey & Bassendowski, 2017). Nursing practice is based on nursing research. Nurses are required to formulate evidence‐based guidelines and establish and implement research and clinical policies and standards. In 2011, the discipline of nursing was established as a national level 1 discipline. Its establishment requires nurses to have solid nursing expertise, and a strong sense of research and research ability (Wang et al., 2015). Nursing scientific research ability refers to the ability to carry out research. It is also used to evaluate the ability of nurses to evaluate, synthesise and implement research results, including mastering the basic knowledge of nursing scientific research, basic scientific research methods, literature retrieval skills, health statistics knowledge, statistical software operation, scientific research paper writing and other abilities (Liu, 2004; McCance et al., 2007).

SNs are senior practitioners of clinical nursing and promoters of evidence‐based nursing practice (Nie et al., 2019). The international literature has no consistent terminology for advanced practice nurses (Jokiniemi et al., 2012). Since the beginning of advanced nursing practice, SNs have been implemented in various healthcare settings and medical specialities and positively impact patient outcomes and access to healthcare services (Coster et al., 2018; Woo et al., 2017). According to the International Council of Nurses (ICN), a CNS is “an advanced practice nurse who provides expert clinical advice and care based on established diagnoses in specialised clinical fields of practice along with a systems approach in practicing as a member of the healthcare team” (ICN, 2020). A CNS is usually at least one master‐ready nurse whose roles include advanced responsibilities, specialisation and extended practice (ICN, 2020). CNSs of different specialities in the United States have profound theoretical knowledge, rich clinical experience and superb clinical skills in specific clinical nursing. CNSs are required to obtain a master's degree in nursing and play an active role as expert advisors to other nurses to enhance the professionalism of the nursing staff (Zhang, Yan, et al., 2020; Zhang, Zhang, et al., 2020). In Denmark, Iceland and Finland, the minimum CNS education is at the master's level (Jokiniemi et al., 2019; Studienævnet for Sundhedsvidenskab, 2019; The Directorate of Health, 2019). In Japan, the training goals of CNS and CN specialised nursing talents are nursing practice experts, but the education level is different. The combination of CNS and master's degree mainly plays a role in nursing practice, consultation and guidance (Meng, 2019). CNSs in Korea are required to complete a postgraduate specialist nursing education program offered by an institution accredited by the Ministry of Health and Welfare (Kang, 2005; Kim et al., 2010). National Health Commission of the People's Republic of China (2017), which 48 points out that, by 2020, a group of SNs will be trained in a planned manner to meet the needs of clinical nursing, develop a team of SNs and improve the level of specialist nursing (Nie et al., 2019). Specialised nurses play an increasingly important role in China's clinical work.

SNs in China must be RNs, with a Junior degree or above, with 3 years or more of nursing work experience and 2 years or more of specialist work experience, completed in 2–7 months. After training and passing the relevant examinations, nurses can obtain the certificate of the relevant SN (Zhao & Sheng, 2019). In developed countries, SNs are mostly positioned as CNSs, and the educational requirements are relatively high. Compared with developed countries, China still has a big gap (Guo et al., 2015). This large gap may be related to the late development of SNs and their training system in China. In China, entry criteria for SNs vary depending on the area of specialist care. Provinces and municipalities have also developed different entry criteria in their SNs training systems, resulting in uneven competencies and low recognition of SNs across the country (Ma et al., 2019; Zhang et al., 2012).

The scientific research level and ability of SNs determine whether they can find, analyse and solve clinical nursing problems from the perspective of speciality and play the role of nursing experts in practice (Mo et al., 2020a, 2020b). In China, SNs undertake the work of specialist outpatient clinics, consultations, teaching and scientific research. According to the survey (Gan et al., 2014; Jing et al., 2016), the scientific research ability of Chinese nurses is at medium and low levels and shows a polarisation trend. The impact of nursing research in China is smaller than that in other countries (Uys et al., 2013). In the past two decades, nurses in Chinese academic institutions and large hospitals have been committed to improving their research ability, such as improving nurses' research awareness, involving nurses in research and improving research productivity (Zhang, Yan, et al., 2020; Zhang, Zhang, et al., 2020). Guangxi Zhuang Autonomous Region is one of the five ethnic minority autonomous regions in China and the only coastal autonomous region in China. Given historical reasons, it lags behind in various aspects and has a low level of economic development. Although researchers in China have investigated the scientific research ability of nurses in a some areas or hospitals, no research has been conducted on the current situation and influencing factors of nursing research ability of SNs in the Guangxi Zhuang Autonomous Region. The present study provides a basis for nursing managers in Guangxi hospitals to develop personalised research training programmes to improve the research capacity of SNs by investigating the current situation and influencing factors of nursing research capacity of SNs in Guangxi Zhuang Autonomous Region.

## 3 METHODS

### 3.1 Design

For data collection, a cross‐sectional questionnaire survey was conducted from March to October 2021. The manuscript was developed using the STROBE checklist for cross‐sectional studies.

### 3.2 Participants

The sample selected Guangxi SNs in 2021, including critical care, emergency care, intravenous therapy care, paediatric care, perioperative care, cancer care, obstetric care, diabetes care, haemodialysis care, wound care, rehabilitation care and geriatric care.

### 3.3 Assessment instruments

The survey consists of two parts: demographic characteristics and a self‐assessment scale of scientific research ability.

#### 3.3.1 Demographic characteristics

We used a self‐designed demographic questionnaire to collect the information of participants, including gender, age, nursing work experience, educational background, professional title, level of working hospital and whether they have published papers as the first author and successfully applied for scientific research topics. These factors were selected based on previous research (Wang & Li, 2020; Wu et al., 2021; Yuan, 2017).

#### 3.3.2 Nursing Research Competence of Nurses Self‐evaluation Scale

The self‐assessment scale designed by Pan and Cheng (2011) was used for assessing the scientific research ability of nursing staff. It has good reliability and validity. The Cronbach's *α* coefficient of this scale was 0.861, and the test–retest reliability was 0.902. The Cronbach's *α* for subscales ranged from 0.655 to 0.760, whereas the RMSEA value in the confirmatory factor analysis was 0.095. The scale has 6 dimensions and 30 items, and responses are scored on a five‐point Likert scale. The dimensions are problem discovery ability (three items), literature review ability (five items), scientific research design ability (five items), scientific research practice ability (six items), data processing ability (five items), and thesis writing ability (six items). The total score ranges from 0 to 120, with higher scores indicating the stronger scientific research ability of nurses. The final scores are categorised as follows: low level (0–40), medium level (41–80) and high level (81–120).

### 3.4 Data collection

A paper version of the questionnaire was distributed to the SNs surveyed between March and October 2021. The two persons responsible for the survey received uniform training to understand fully the purpose of the survey and the meaning of each question in the questionnaire. All participants were required to sign an informed consent form before answering the questionnaire. The respondents completed the questionnaire independently and returned on the spot after completing the questionnaire. After the investigation, two investigators were responsible for checking the questionnaires and eliminating the questionnaires with missing items or logical contradictions. All personal information was encoded, and participants were made anonymous using a reference number system. Their data were then stored safely in an encrypted database. For the sample size, the recommendations of between five and ten subjects for each item on the questionnaire were considered (Zhan et al., 2020). Considering that 10% are invalid questionnaires, a minimum of 209 SNs was required. Finally, 665 SNs completed the questionnaire, and 13 invalid questionnaires were excluded. These invalid questionnaires were mainly because of incomplete questionnaires. A total of 652 valid questionnaires were recovered, with a completion rate of 98.05%.

### 3.5 Statistical analysis

The obtained data were entered and analysed using statistical analysis software SPSS version 17.0. Two research assistants rechecked the data, and the questionnaires that did not meet the inclusion criteria were excluded from the analysis. The normality tests were used to observe whether the data conform to normal distribution. The median (interquartile range) was used to describe data because of their skewed distribution. Classification data were calculated by frequency and proportion. Wilcoxon rank‐sum test or Kruskal–Wallis *H*‐test was used to determine the difference in median values among different groups. The score of scientific research ability was set as the dependent variable, and it is divided into low, medium and high levels. Finally, we performed ordinal logistic regression analysis using all potential factors identified early in univariate analysis. For all statistical tests, the significance level was set at 0.05.

### 3.6 Ethics

All participants agreed and voluntarily participated in the survey, and the investigators ensured the anonymity and confidentiality of the data. The study was reviewed and approved by the university research ethics review committee.

## 4 RESULTS

Six hundred and fifty‐two SNs are from 14 prefecture‐level cities in Guangxi Zhuang Autonomous Region, including Nanning City (*n* = 153), Baise City (*n* = 67), Hechi City (*n* = 41), Guilin City (*n* = 61), Liuzhou City (*n* = 86), Yulin City (*n* = 38), Hezhou City (*n* = 17), Wuzhou City (*n* = 38), Guigang City (*n* = 26), Laibin City (*n* = 26), Chongzuo City (*n* = 16), Qinzhou City (*n* = 45), Fangchenggang City (*n* = 15) and Beihai City (*n* = 23).

### 4.1 Participant characteristics

The characteristics of the respondents are presented in Table 1. Approximately 97.1% (*n* = 633) of the participants were women, and most of the SNs were aged 30–39 years (*n* = 478, 73.3%). SNs working for 10–14 years comprised the largest group (*n* = 282, 43.3%) and 346 participants were from tertiary hospitals (*n* = 54.1%). Most SNs have undergraduate degree (*n* = 513, 78.7%) and only a few have a master's degree or above (*n* = 9, 1.4%). More than half of the SNs have primary professional titles (*n* = 346, 53.1%) and most of them have not published papers as the first author (*n* = 530, 81.3%) and completed projects (*n* = 631, 96.8%).

**TABLE 1:** Demographic characteristics of participants (*N* = 652).

Variate	*N*	%
Gender		
Male	19	2.9
Female	633	97.1
Age (years)		
20–29	137	21.0
30–39	478	73.3
≥40	37	5.7
Nursing work experience (years)		
1–4	6	0.9
5–9	228	35.0
10–14	282	43.3
15–19	108	16.6
≥20	28	4.3
Educational background		
Junior college or below	130	19.9
Undergraduate	513	78.7
Master degree or above	9	1.4
Professional title		
Junior title	346	53.1
Intermediate title	295	45.2
Senior title	11	1.7
Level of working hospital		
Tertiary	353	54.1
Secondary	299	45.9
Whether you ever published a paper as the first author		
No	530	81.3
Yes	122	18.7
Whether you successfully applied for scientific research projects		
No	631	96.8
Yes	21	3.2

### 4.2 Score of scientific research ability of SNs

Table 2 shows that the total score of scientific research ability of specialised nurses is low, and the median score is 31 (interquartile range: 19–41), among which the score of scientific research design ability is the lowest. The scores were divided into three levels: low (*n* = 488, 74.8%), medium (*n* = 156, 23.9%) and high (*n* = 8, 1.2%).Table 3 provides the responses to each question.

**TABLE 2:** Dimensions and total scores of nurses' scientific research ability self‐assessment scale.

Variate	Score range	Score
Problem finding ability	0–12	5.00 (3.00, 6.00)
Literature review ability	0–20	6.00 (5.00, 8.00)
Scientific research and design ability	0–20	4.00 (1.00, 5.00)
Scientific research practice ability	0–24	6.00 (3.00, 8.00)
Data processing capability	0–20	5.00 (1.00, 6.00)
Thesis writing ability	0–24	6.00 (2.00, 8.00)
Total score	0–120	31.00 (19.00, 41.00)

**TABLE 3:** Responses of the nurses' scientific research ability self‐rating scale.

	Frequency				
Item	Hard to do it, *n* (%)	Rarely do it, *n* (%)	Generally do it, *n* (%)	Often do it, *n* (%)	Totally do it, *n* (%)
Q1: In nursing work, problems that can be used as reference for research are found	121 (18.6)	368 (56.4)	125 (19.2)	34 (5.2)	4 (0.6)
Q2: When encountering problems in nursing work, be able to put forward ideas to solve the problems	9 (1.4)	211 (32.4)	306 (46.9)	116 (17.8)	10 (1.5)
Q3: When encountering problems in nursing work, we often look for theoretical basis	13 (2.0)	264 (40.5)	242 (37.1)	123 (18.9)	10 (1.5)
Q4: Often consult relevant professional materials manually	32 (4.9)	312 (47.9)	194 (29.8)	106 (16.3)	8 (1.2)
Q5: Often search relevant professional information through the Internet	9 (1.4)	218 (33.4)	242 (37.1)	165 (25.3)	18 (2.8)
Q6: Proficient in using Chinese database	101 (15.5)	344 (52.8)	146 (22.4)	50 (7.7)	11 (1.7)
Q7: With the help of auxiliary tools, be able to read nursing foreign literature	228 (35.0)	288 (44.2)	114 (17.5)	20 (3.1)	2 (0.3)
Q8: Objective evaluation of Nursing Research Report	183 (28.1)	349 (53.5)	105 (16.1)	15 (2.3)	0 (0.0)
Q9: Use the formula to calculate the sample size	289 (44.3)	273 (41.9)	77 (11.8)	12 (1.8)	1 (0.2)
Q10: Be able to determine the inclusion scope of the research object	178 (27.3)	337 (51.7)	118 (18.1)	17 (2.6)	2 (0.3)
Q11: Determine the observation indicators of the research project	223 (34.2)	326 (50.0)	90 (13.8)	10 (1.5)	3 (0.5)
Q12: According to the research purpose, qualitative, quantitative or a combination of the two research methods are selected	254 (39.0)	312 (47.9)	75 (11.5)	11 (1.7)	0 (0.0)
Q13: Determine the evaluation criteria for the research results	240 (36.8)	309 (47.4)	87 (13.3)	14 (2.2)	2 (0.3)
Q14: Formulate nursing research plan	224 (34.4)	311 (47.7)	95 (14.6)	19 (2.9)	3 (0.5)
Q15: In scientific research practice, ethical issues can be considered	169 (25.9)	285 (43.7)	156 (23.9)	32 (4.9)	10 (1.5)
Q16: In scientific research practice, interference factors can be considered	156 (23.9)	317 (48.6)	149 (22.9)	28 (4.3)	2 (0.03)
Q17: When the scientific research practice is inconsistent with the design, the research scheme can be adjusted in time	186 (28.5)	306 (46.9)	138 (21.2)	18 (2.8)	4 (0.6)
Q18: Skilfully observe and record research related information	168 (25.8)	320 (49.1)	138 (21.2)	22 (3.4)	4 (0.6)
Q19: Skilfully interview research subjects	184 (28.2)	315 (48.3)	125 (19.2)	28 (4.3)	0 (0.0)
Q20: Be able to make common statistical charts	177 (27.2)	326 (50.0)	122 (18.7)	22 (3.4)	4 (0.6)
Q21: Capable of statistical description	221 (33.9)	319 (48.9)	97 (14.9)	12 (1.8)	3 (0.5)
Q22: Select appropriate statistical analysis methods	223 (34.2)	320 (49.1)	95 (14.6)	11 (1.7)	3 (0.5)
Q23: Correct interpretation of statistical results	196 (30.1)	327 (50.2)	114 (17.5)	10 (1.5)	5 (0.8)
Q24: Analysis of qualitative research data	257 (39.4)	315 (48.3)	70 (10.7)	9 (1.4)	1 (0.2)
Q25: Standardise the writing of the preface of the paper	198 (30.4)	311 (47.7)	121 (18.6)	21 (3.2)	1 (0.2)
Q26: Standardised writing of abstracts of papers	189 (29.0)	305 (46.8)	135 (20.7)	21 (3.2)	2 (0.3)
Q27: Standardise and refine the key words of the paper	152 (23.3)	314 (48.2)	154 (23.6)	28 (4.3)	4 (0.6)
Q28: Research results of standardised writing papers	211 (32.4)	322 (49.4)	102 (15.6)	15 (2.3)	2 (0.3)
Q29: Discussion on standardised writing of papers	199 (30.5)	330 (50.6)	109 (16.7)	12 (1.8)	2 (0.3)
Q30: References for standardised writing papers	172 (26.4)	300 (46.0)	147 (22.5)	26 (4.00)	7 (1.1)

### 4.3 Influencing factors of scientific research ability of SNs

In Table 4, the univariate analysis findings indicated statistically significant differences in the scores of scientific research ability in terms of the number of years of nursing work experience, educational background and level of working hospital whether the first author has published paper and successfully applied for scientific research topics (*p* < 0.05). The model conforms to the test of parallel lines (*χ* ^2^ = 4.655, *p* = 0.863), indicating that the regression equations are parallel to each other and can be analysed using ordinal logistic regression. However, ordinal logistic regression analysis showed that educational background, level of working hospital, whether the first author had published papers, and successful application for scientific research topics were the factors affecting the score of scientific research ability. The variable “years of nursing experience” was eliminated (Table 5).

**TABLE 4:** Univariate analysis of nurses' scientific research ability.

Item	*n*	Scores	*χ* ^2^/*Z*	*p*
Gender			−1.138	0.255
Male	19	36.00 (21.00, 52.00)		
Female	633	31.00 (19.00, 40.00)		
Age (years)			0.472	0.790
20–29	137	32.00 (19.00, 42.00)		
30–39	478	31.00 (19.00, 40.00)		
≥40	37	30.00 (16.50, 44.00)		
Nursing work experience (years)			11.915	0.018
1–4	6	58.50 (30.50, 73.25)		
5–9	228	31.00 (19.00, 40.00)		
10–14	282	30.00 (18.00, 39.00)		
15–19	108	34.00 (23.25, 47.00)		
≥20	28	28.50 (16.25, 40.25)		
Educational background			21.628	<0.001
Junior college or below	130	27.50 (16.75, 36.00)		
Undergraduate	513	31.00 (19.50, 41.00)		
Master degree or above	9	67.00 (50.50, 79.50)		
Professional title			3.206	0.201
Junior title	346	30.00 (18.00, 39.25)		
Intermediate title	295	31.00 (20.00, 42.00)		
Senior title	11	31.00 (28.00, 61.00)		
Level of working hospital			−5.187	<0.001
Tertiary	353	34.00 (22.00, 45.00)		
Secondary	299	29.00 (16.00, 37.00)		
Whether you ever published a paper as the first author			−6.079	<0.001
No	530	30.00 (17.00, 38.00)		
Yes	122	38.00 (29.00, 51.00)		
Whether you successfully applied for scientific research projects			−4.428	<0.001
No	631	30.00 (19.00, 40.00)		
Yes	21	53.00 (33.50, 72.00)		

**TABLE 5:** The result of logistic regression analysis of nurses' scientific research ability influencing factors.

Variables	*B*	SE	Wald *χ* ^2^	*p*	95% CI
Nursing work experience (years)					
1–4	0.326	1.096	0.088	0.766	−1.823 to 2.475
5–9	0.267	0.507	0.277	0.599	−0.726 to 1.256
10–14	0.177	0.499	0.126	0.723	−0.801 to 1.155
15–19	0.418	0.515	0.659	0.417	−0.592 to 1.429
≥20	Reference	Reference	Reference	Reference	Reference
Educational background					
Junior college or below	−3.165	0.855	13.687	<0.001	−4.842 to −1.488
Undergraduate	−2.938	0.824	12.719	<0.001	−4.552 to −1.323
Master degree or above	Reference	Reference	Reference	Reference	Reference
Level of working hospital					
Tertiary	0.413	0.202	4.182	0.041	0.017–0.810
Secondary	Reference	Reference	Reference	Reference	Reference
Whether you ever published a paper as the first author					
No	−0.791	0.240	10.813	0.001	−1.261 to −0.320
Yes	Reference	Reference	Reference	Reference	Reference
Whether you successfully applied for scientific research projects					
No	−1.338	0.478	7.830	0.005	−2.274 to −0.410
Yes	Reference	Reference	Reference	Reference	Reference

## 5 DISCUSSION

### 5.1 Current situation of scientific research ability of SNs

The scientific research ability of nurses is an important factor used to measure the quality of nursing education (Zhang et al., 2012). This study shows that the scientific research ability of SNs is generally at a low level, consistent with the investigation of Guo and Zhang (2016) and Lv et al. (2018). This finding may be because of the lack of scientific research knowledge and methods, especially knowledge related to scientific research design and data processing (Kelly et al., 2016). In the present work, the problem‐finding ability is the highest, and the scientific research and design ability is the lowest, consistent with the research results of Wang and Li (2020) and Wu et al. (2021). Most of the nurses participating in the training course are excellent talents of the hospital, have rich clinical work experience and can actively think and find clinical nursing problems in clinical nursing work. However, they focus more on specialist nursing knowledge and skills and have not received systematic training in nursing research theories and methods. Deficiencies exist in correctly carrying out research design. Therefore, improving the scientific research ability of SNs and carrying out targeted intervention is a problem that needs to be addressed.

### 5.2 Influencing factors of scientific research ability of SNs

#### 5.2.1 Education is one of the factors affecting nurses' scientific research ability

This study shows that the higher the education is, the stronger the scientific research ability will be. SNs with graduate education have the strongest scientific research ability, consistent with the results of Yuan (2017), Huang et al. (2012), Cheng et al. (2010), and Robichaud‐Ekstrand (2016). However, in Guangxi Zhuang Autonomous Region, the proportion of SNs with undergraduate education is the largest, accounting for 78.7%, whereas the proportion of SNs with graduate education is the smallest, accounting for only 1.7%, which relates to China's early nursing education. On the one hand, China's SNs have low initial education levels and have not undergone systematic scientific research training, and their scientific research foundation is relatively weak. Furthermore, the general view of clinical specialists is that research can only be carried out by people with advanced degrees. Given the complexity and tediousness of the research process, nurses with ordinary education cannot participate, and the application of scientific research knowledge to clinical practice is beyond their scope (Berthelsen & Hølge‐Hazelton, 2015; Silka et al., 2012). On the other hand, short‐term scientific research training is unlikely to make a qualitative change in the scientific research ability of SNs. Although hospitals or schools will set up relevant courses on nursing research, some problems persist, such as short class hours, unsystematic teaching content and incomplete knowledge structure. Furthermore, few nurses with a doctoral degree who have received academic training are engaged in nursing practice (Hølge‐Hazelton et al., 2016). They are usually employed by universities and educational institutions, leaving the challenging task of creating a nursing research culture for clinical nurses and leaders with limited research ability. Since 1970, nursing research in the United States has made great progress because they have a high starting point of nursing education and nurses can receive relevant knowledge of nursing research during school (Dobratz et al., 2012). Within Denmark, Iceland and Finland, the minimum CNS education is at the master's level, which includes 90 to 120 European Credit Transfer System credits (Jokiniemi et al., 2019; Studienævnet for Sundhedsvidenskab, 2019). The main factors behind the uneven development of nursing science between different countries are the differences in physical and human resources between countries and the importance attached to building the disciplinary field of nursing. To support the development of global nursing science, leaders should assess contextual support for nursing researchers and strategically plan for the further development of the infrastructure needed to promote the health of people globally and develop strategic initiatives to improve environmental support in their countries. Hospital managers are hoped to pay attention to the development of the nursing profession, actively encouraging nurses to participate in relevant continuing education, learn appropriate scientific research knowledge, accumulate relevant scientific research experience, apply the scientific research knowledge learned to clinical work and provide the basis and guarantee of scientific research for clinical work.

#### 5.2.2 Level of working hospital is one of the factors affecting nurses' scientific research ability

In China, the health administrative department will conduct the hierarchical management of hospitals according to their functions, tasks, facility conditions, technical construction, medical service quality and scientific management level. A tertiary hospital is a medical prevention technology centre with comprehensive medical, teaching and scientific research capabilities. Its main functions are to provide specialised medical services, solve critical and difficult diseases and accept referrals from lower‐level hospitals, provide business and technical guidance to lower‐level hospitals and train people, completely cultivate the teaching of various senior medical professionals and undertake the task of scientific research projects above the provincial level, participating in and guiding primary and secondary prevention work. Compared with secondary hospitals, specialist nurses working in tertiary hospitals have stronger scientific research ability. The reason may be that, in China, the construction of nursing scientific research ability has become an important management goal, especially in tertiary hospitals and university‐affiliated hospitals (Li et al., 2009). Tertiary hospitals pay more attention to nursing research and nursing innovation than secondary hospitals because the evaluation standards of China's tertiary hospitals stipulate that some systems and methods should encourage all staff to participate in scientific research; promote the transformation of scientific research achievements into clinical application; and provide appropriate funds, conditions, facilities and personnel support (National Health Commission of the People's Republic of China, 2020). Therefore, tertiary hospitals will encourage clinical nurses to learn nursing scientific research knowledge in various ways, build a good scientific research platform for nurses and provide a supportive scientific research environment. Tertiary hospitals also establish nursing scientific research management systems and increase investment in scientific research funds. They also give sufficient financial and spiritual rewards to nurses who obtained scientific research achievements to make them proud of scientific research achievements, improve the enthusiasm of clinical nurses to participate in scientific research and continuously stimulate nurses' interest in scientific research through reasonable ways to promote more clinical nurses to participate in nursing scientific research (Jiang et al., 2018).

#### 5.2.3 Whether to publish papers and successfully apply for scientific research projects are also factors affecting the scientific research ability of SNs

This study shows the higher score of scientific research ability of clinical SNs who have published papers and successfully applied for scientific research topics, consistent with the research results of Jin and Liu (2015) and Shang et al. (2018). The reason is that, in the process of publishing papers and applying for topics, nurses need to consult a large number of nursing journals, obtain extensive information and timely understand the new trends of nursing development at home and abroad, which will expand their clinical scientific research thinking and accumulate a certain scientific research foundation. Furthermore, in writing academic papers and applying for scientific research topics, clinical SNs can continuously learn scientific research knowledge and finally improve their scientific research ability through literature review, data processing and paper writing. However, to publish papers and declare topics, clinical SNs should also have certain research abilities (Corchon et al., 2010) and actively participate in the research process (Walker, 2006), which requires high‐quality research training and guidance (Bournes, 2013) and learning ability. Clinical nursing managers should actively create a good scientific research competition atmosphere, foster teamwork, encourage academic exchanges, strengthen the training of clinical nurses' scientific research paper writing and application topics, improve the quality of nursing scientific research training, broaden the scientific research training platform, advocate personalised training methods and encourage clinical nurses to participate in nursing scientific research (Zhu et al., 2020).

## 6 LIMITATIONS

The findings of this regional survey should be interpreted cautiously because of the following limitations. Firstly, this study was conducted in the Guangxi Zhuang Autonomous Region, with different numbers of SNs in each prefecture‐level city and no additional data from other provinces. Therefore, the results cannot be generalised to all Chinese SNs. Secondly, this study is a self‐report survey method, which may lead to inflated or underestimated scores on the measurement scale, and possible inherent bias cannot be ruled out. Finally, only the SNs in 2021 were selected for the survey, and SNs from other years and disciplines will be included in the survey in the future.

## 7 CONCLUSION

The overall level of scientific research ability of SNs in Guangxi Zhuang Autonomous Region is low, which is related to influencing factors, such as educational background, level of working hospital, whether they have published papers as the first author and successful application for scientific research projects. Chinese SNs have rich clinical experience but lack theoretical basis and training of relevant scientific research knowledge. Thus, hospitals and clinical nursing managers should actively create a good scientific research competition atmosphere, advocate teamwork, encourage academic exchanges, broaden the scientific research training platform, strengthen scientific research training for clinical nurses and encourage clinical nurses to participate in nursing scientific research. They should also advocate clinical speciality nurses to enrich their nursing scientific research knowledge, improve scientific research awareness, cultivate scientific research innovation ability and better solve clinical problems with scientific research knowledge to promote the development of nursing.

## AUTHOR CONTRIBUTIONS

Ziwei Huang, Yiping Wei, Yi Lei: Study design. Yuangfang Liu, Xiaomei Chen, Yuangfang Liu: Data collection. Ziwei Huang, Yuangfang Liu: Data analysis. Ziwei Huang: Manuscript writing.

## FUNDING INFORMATION

This study was supported by Education Department of Guangxi Zhuang Autonomous Region (Grant Number: 2020JG147) and Innovation Project of Guangxi Graduate Education (Grant Number: JGY2020051).

## CONFLICT OF INTEREST STATEMENT

The authors declare no conflicts of interest.

## ETHICS STATEMENT

This study maintains the anonymity of all the participants, who can opt whether or not to participate. Formal written consent was obtained from all participants. We further guaranteed that the identity of the participants will not be disclosed and that their answers will be confidential. Ethical approval for the project was gained from the ethics committees of the Guangxi Medical University.

## Data Availability

The data sets generated for this review are available on reasonable request to the corresponding author.
